# Charge Carrier Density in Organic Semiconductors Modulates the Effective Capacitance: A Unified View of Electrolyte Gated Organic Transistors

**DOI:** 10.1002/adma.202410940

**Published:** 2024-10-15

**Authors:** Rian Zanotti, Matteo Sensi, Marcello Berto, Alessandro Paradisi, Michele Bianchi, Pierpaolo Greco, Carlo Augusto Bortolotti, Michele Di Lauro, Fabio Biscarini

**Affiliations:** ^1^ Dipartimento di Scienze della Vita – Università di Modena e Reggio Emilia Via Campi 103 Modena 41125 Italy; ^2^ Dipartimento di Fisica, Informatica e Matematica Università di Modena e Reggio Emilia Via Campi 103 Modena 41125 Italy; ^3^ Center for Translational Neurophysiology of Speech and Communication (CTNSC) – Istituto Italiano di Tecnologia Via Fossato di Mortara 17–19 Ferrara 44100 Italy; ^4^ Sezione di Fisiologia Umana Università di Ferrara Via Fossato di Mortara 19 Ferrara 44100 Italy

**Keywords:** density of states, EGOFET, OECT, transconductance, transfer curve

## Abstract

A framework for electrolyte‐gated organic transistors (EGOTs) that unifies the view of interfacial capacitive coupling of electrolyte‐gated organic field‐effect transistors (EGOFETs) with the volumetric capacitive coupling in organic electrochemical transistors (OECTs) is proposed. The EGOT effective capacitance arises from in‐series capacitances of the electrolyte/gate electrode and electrolyte/channel interfaces, and the chemical capacitance of the organic semiconductor channel whose weight with respect to the interfacial capacitance is modulated by the charge carrier density, hence by the gate voltage. The expression for chemical capacitance is derived from the DOS of the organic semiconductor, which it is assumed to exhibit exponential energy disorder in the HOMO‐LUMO gap. The analytical expression of the EGOT current is assessed on experimental data and shown to accurately predict the shape of the whole transfer curve of an EGOT thus allowing to extract accurate values for the switch‐on voltage and the interfacial transconductance, without assumptions on specific response regime and, in OECT, without invoking the volumetric capacitance. Interestingly, the EGOT model recovers EGOFET and OECT as limit cases and, in the latter case, explicitly represents the volumetric capacitance in terms of the energy disorder and the bandgap of the organic semiconductor.

## Graphical abstract

A framework for electrolyte gated organic transistors (EGOTs) that unifies electrolyte‐gated organic field‐effect transistors (EGOFETs) with organic electrochemical transistors (OECTs) is proposed. The effective capacitance that drives the device response arises from in series capacitances of device interfaces and the chemical capacitance of the organic semiconductor. The model predicts the shape of the transfer curve of EGOTs (p‐/n‐type and ambipolar).

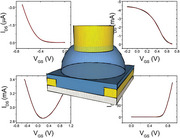

## 1 Introduction

Electrolyte gated organic transistors (EGOT) emerged as ultrasensitive sensors and transducers of chemical and electrical signals, both in vitro and in vivo.^[^ ^1^, ^2^, ^3^, ^4^ ^]^ Their outstanding performance in terms of limit of detection (LOD) and sensitivity stems from their large transconductance that was ascribed, in analogy with ion‐sensitive field‐effect transistors (ISFET), to the interfacial capacitance between the electrolyte and the device.^[^ ^5^, ^6^, ^7^ ^]^ In the most common device architecture, the organic semiconductor channel is immersed in the same electrolyte playing the role of the gate dielectric. The possibility that the electrolyte penetrates the semiconductor channel was overlooked, privileging instead the explanation based on the capacitance of the electrical double layers created at the interfaces between the gate and the electrolyte and between the channel and the electrolyte.^[^ ^2^, ^7^ ^]^ This interpretation assumes that charge accumulation in the semiconductor channel can be modelled as a parallel plate capacitor, in analogy with models developed for thin‐film field‐effect transistors.^[^ ^8^, ^9^ ^]^ These devices with interfacial capacitive coupling were termed electrolyte‐gated organic field effect transistors (EGOFETs). Yet, the purely interfacial capacitive model fails to quantitatively describe some features of their response, such as non‐linear transfer curves and subthreshold behaviour, which are relevant especially in sensing operations.

Reported evidence of EGOFET response suggests that percolation of the electrolyte solution through the semiconductor channel occurs,^[^ ^10^, ^11^, ^12^, ^13^ ^]^ which would make ions to strongly interact with the organic semiconductor across the thickness of the thin film channel. This observation heralds the so‐called chemical capacitance (termed quantum capacitance in the context of 2D materials) as a potential player in the gating of EGOFETs.

It is also widely recognized that in the case of organic electrochemical transistors (OECTs) working in depletion, as those based on polymeric conductors like Poly(3,4‐ethylenedioxythiophene) polystyrene sulfonate (PEDOT:PSS), the electrolyte penetrates the channel, giving rise to mixed electronic‐ionic conductivity and to the modulation of the electronic current by means of ion currents controlled by the gate bias.^[^ ^14^ ^]^ In this case, the interfacial capacitance of the organic channel at the electrolyte interface was substituted with the concept of volumetric capacitance.^[^ ^9^, ^15^, ^16^, ^17^ ^]^ If, on the one hand, introducing this ad hoc property reconducts the observed response to the well‐known equations of thin film transistors, it creates some issues on the actual mechanism of current modulation with ions. The rising question is whether the volumetric capacitance is needed to describe devices which are based on the same family of organic semiconductors as in EGOFETs, the only difference being the initial doping level of the active material. Indeed, experiments conducted on large‐volume PEDOT:PSS slabs indicate that there is a linear correlation between the capacitance of the PEDOT:PSS (extracted from electrochemical impedance spectroscopy) and the electroactive surface area (ESA), as both scale with the volume of PEDOT:PSS,^[^ ^16^ ^]^ thus hinting to the central role of a thickness‐invariant areal capacitance when normalized to the ESA.

## 2 Density of States of the Organic Semiconductor

In this work, we address the problem of establishing a coherent description of both EGOFETs and OECTs as EGOT, by introducing the chemical capacitance in the description of the device. This allows us to formulate a model that includes in series interfacial and chemical capacitances, the latter being dependent on the charge carrier density and hence on the gate voltage *V_GS_*. We first formulate the general expression for the chemical capacitance of the organic semiconductor based on the density of states (DOS), then work out a general expression for the charge carrier density in an EGOT channel. We show that the charge carrier density is, in general, a non‐linear function of the gate voltage bias. In the acquisition of the EGOT transfer curve, the gate voltage modulates the weight of the chemical capacitance within the effective capacitance of the EGOT. We identify the regime where the chemical capacitance is so large that can be disregarded in favor of the description based on the sole interfacial capacitance, viz. the EGOFET. In *p*‐type semiconductors, this occurrence is attained for channels with a low energy disorder and high density of states in the DOS region of interest. Interestingly, the film thickness, which appears as a scaling variable of the problem, cancels out exactly in the regime where the effective capacitance is purely interfacial. In the opposite limit case of large energy disorder and small bandgap, instead, the effective capacitance is dominated by the chemical capacitance that scales linearly with the film thickness, viz. the OECT. Our model shows that without introducing a phenomenological volumetric capacitance, the latter emerges properly in the OECT limit case and is related to the electronic structure of the channel.

**Figure 1:**
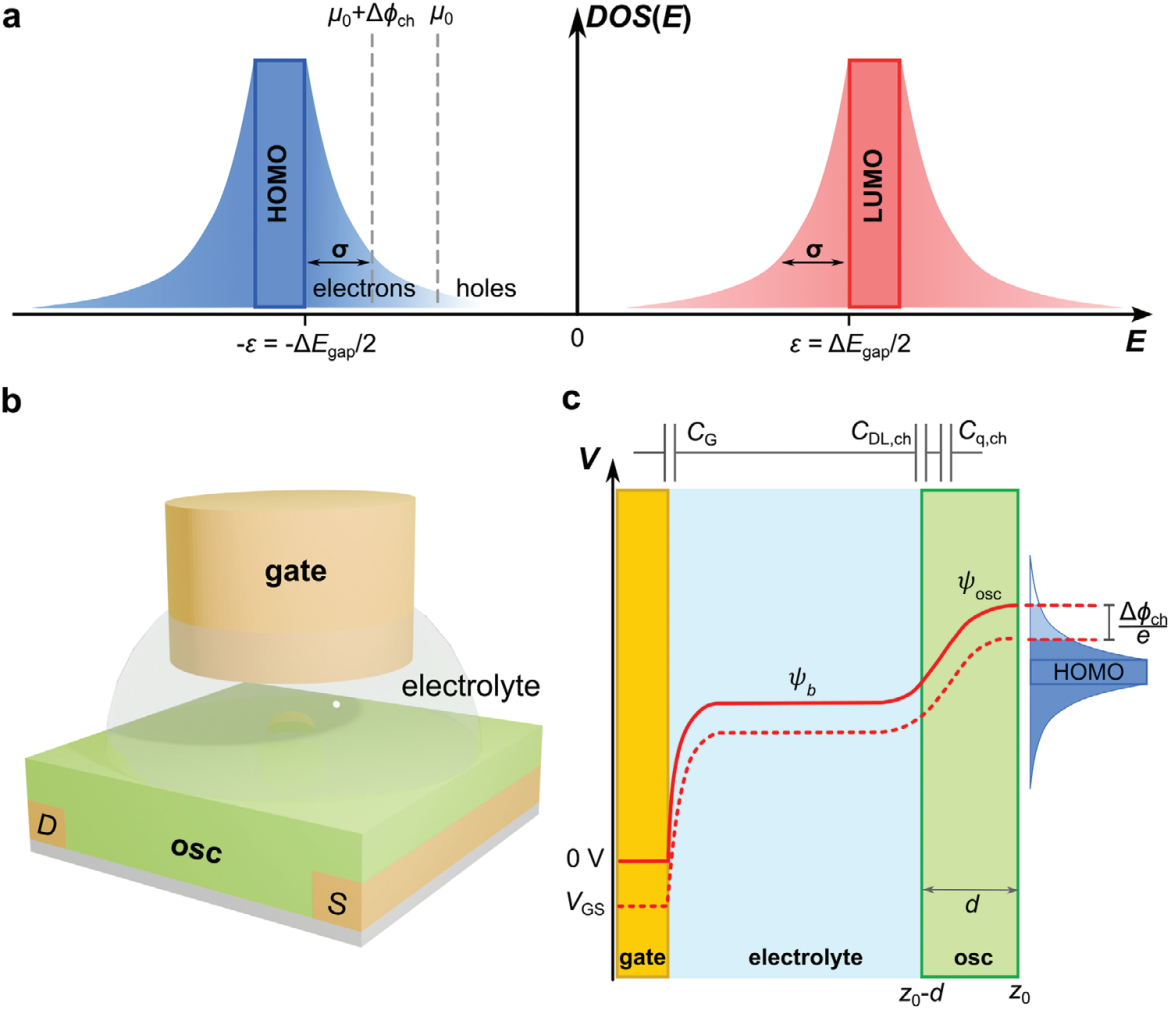
a) Schematic representation of the Density of States (DOS) versus electronic level energy *E* in the organic semiconductor. The HOMO and LUMO bands are depicted. The DOS tails that protrude from the band edge of the HOMO and LUMO narrow bands into the gap are described as exponential functions. The energy disorder parameter σ is their decay rate. b) Schematic representation of an EGOT. c) Potential profile of the EGOT with a metal gate electrode, in the hypothesis of constant potential along the channel.

We first discuss the DOS of an organic semiconductor as a function of energy, as schematically depicted in **Figure** **1**a. The energy properties will be expressed from now on in eV. Here, *μ*_0_ is the electrochemical potential of the semiconductor. The zero of the energy axis is set at the center of the bandgap Δ*E*_gap_ = 2ɛ which makes the HOMO and LUMO DOS edges to be ɛ_0*H*_ = − ɛ and ɛ_0*L*_ = ɛ. The electrochemical potential ranges as − ɛ ≤ μ_0_ ≤ ɛ, in the specific case of Figure 1a, hence *μ*_0_ < 0 implies that the HOMO band tail is populated with hole carriers. Δϕ_ch_ is the shift of the channel electrochemical potential upon gating. In the case of a spin‐cast organic semiconductor thin film, we assume that the channel is molecularly and energetically disordered, so we describe it as a 3D semiconductor material with low doping level. For these organic materials, the DOS of HOMO and LUMO narrow bands was described by a Gaussian distribution function.^[^ ^18^, ^19^ ^]^ Here, we surrogate it with an exponential function to facilitate the analytical treatment.^[^ ^20^, ^21^, ^22^, ^23^ ^]^ Thus, we adopt the functional form of the relevant portion of the whole DOS as the exponential tail protruding in the HOMO‐LUMO bandgap. The energy disorder parameter *σ* accounts for the decay rate of the tails of the DOS above the HOMO band upper edge ɛ_0*H*_ for *p*‐type organic semiconductors (hole carriers), or below the LUMO band lower edge ɛ_0*L*_ for *n*‐type organic semiconductors (electron carriers). For the HOMO band it reads $DOS\;\left(E,\varepsilon_{0H}\right)=c_{n}\;\cdot exp\left(-\frac{E-\varepsilon_{0H}}{\sigma }\right)$, while for the LUMO band is $\mathrm{DOS}\;\left(E,\varepsilon_{0L}\right)=c_{n}\cdot \exp\left(\frac{E-\varepsilon_{0L}}{\sigma }\right).$ The normalization constant *c*_n_ has physical dimensions (energy volume)^−1^, so we express the total DOS in the bandgap as:

(1) $$\begin{array}{c} DOS\;\left(E\right)=c_{n}\;\cdot \;\left\{exp\left(-\frac{\varepsilon +E}{\sigma }\right)+exp\left(-\frac{\varepsilon -E}{\sigma }\right)\right\}\\ =2c_{n}\cdot exp\left(-\frac{\varepsilon }{\sigma }\right)cosh\left(\frac{E}{\sigma }\right) \end{array}$$

Equation (1) holds in the bandgap [−ɛ; ɛ]. We will assume that, in general, the energy disorder is close to the band edges, thus ɛ ≫ σ. The normalization constant *c_n_* is obtained by introducing 2*n*_max_ as the total number of available states per unit volume and energy for the charge carriers:

(2) $$\;n_{\max}=c_{n}\;exp\left(-\frac{\varepsilon }{\sigma }\right)\int_{-\varepsilon }^{\varepsilon }dE\cdot cosh\;\left(\frac{E}{\sigma }\right)=c_{n}\;\sigma \left[1-exp\left(-\frac{2\varepsilon }{\sigma }\right)\right]$$

which yields $c_{n}=\frac{1}{\left[1-exp(-\;\frac{2\varepsilon }{\sigma })\right]}\;\frac{n_{\max}}{\sigma }$. For large bandgap with respect to, *n*_max_ ≈ *c_n_* σ.

Upon these premises, the channel of an EGOT with the switch‐on voltage (*V*_T_ ) = 0 V corresponds to μ_0_ = 0 *eV* which implies that there are no charge carriers in the semiconductor channel. In the absence of the gate voltage, μ_0_ is the negative of the voltage *V*_T_, ^[^ ^24^ ^]^ μ_0_ = −*eV_T_*. When *V*_T_ > 0, holes populate the HOMO tail; when *V_T_* < 0, there is an excess of electrons in the LUMO tail. When the gate potential is turned on, then the electrochemical potential becomes μ_0_ = *e*(*V*_GS_ − *V*_T_). Hence, we identify *V*_GS_ = *V*_T_ as the voltage yielding charge neutrality, else an equal density of charge carriers of opposite sign in the channel. The voltage *V*_T_ should then be regarded as a flat‐band or switch‐on potential, rather than a threshold voltage related to a specific current response.

In Figure 1b we show a schematic drawing of an EGOT, in a top gate architecture immersed in the electrolyte. There is no insulation layer between the electrolyte and the semiconductor channel, hence the electrolyte can percolate the semiconductor thin film. Figure 1c shows the potential profile across the EGOT as a function of the distance z. Two scenarios, one with *V*_GS_ = 0 V and one with a negative electric potential applied to the gate electrode, are depicted. The electrolyte occupies the volume from z = 0 to the distance *z*_0_ – *d*, where *z*_0_ is the coordinate of the substrate where the organic semiconductor thin film is cast. We identify the thickness *d* of the organic semiconductor thin film as its mean height, ignoring the local spatially correlated roughness. The potential of the gate varies exponentially at the gate electrolyte interface from *V*_GS_ to the electrolyte potential ψ_*b*_(*V*_GS_). The potential of the channel ψ_*OSC*_ changes with z both at the electrolyte/channel interface and inside the semiconductor, with the boundary conditions ψ_*OSC*_ (*V*_GS_; *z* ≈ *z*_0_) = *V*_T_. The charge carrier variation due to the applied gate potential will induce a shift Δϕ_*ch*_ of the electrochemical potential of the organic semiconductor according to the DOS of the organic semiconductor. As schematized on the right of Figure 1c, it will shift down the potential of the organic semiconductor, thus increasing the density of positive charge carriers in the HOMO tail. Once the voltage *V*_GS_ is fixed by the source channel of the source measurement unit, the electrolyte potential ψ_*b*_(*V*_GS_) will shift causing a change in the voltage drop at the gate/electrolyte interface and the electrolyte/channel. The former is expressed by the areal gate capacitance *C*_G_, the latter by the channel capacitance given by the in‐series interfacial channel capacitance and chemical capacitance.

## 3 Charge Carriers Density

The charge accumulated at the gate electrode/electrolyte interface is *Q*_G_ = *A*_G_C_G_[*V*_GS_ − ψ_*b*_(*V*_GS_)]. Electroneutrality in the EGOT holds for any *V*_GS_ value, hence *Q*_G_ must be compensated by the charge *Q*_ch_ accumulated at the channel, hence *Q*_G_ = *A*_G_ *C*_G_ [*V*_GS_ − ψ_*b*_] = *A*_Ch_ *e*Δ*n* = − *Q*_ch_. Therefore, one obtains the electrolyte potential as

(3) $$\psi_{b}\;\left(V_{\mathrm{GS}}\right)=V_{\mathrm{GS}}-\frac{A_{\mathrm{Ch}}\Delta n\cdot e}{A_{G}C_{G}}$$

Here the gate and channel geometrical areas are *A*_G_ and *A*_ch_, the elementary charge is *e*, and the charge carrier population is expressed by the areal density Δ*n*. For holes Δ*n* ≤ 0, and for electrons Δ*n* ≥ 0. Then, we follow the procedure used to describe the EGT based on reduced graphene oxide (rGO).^[^ ^25^, ^26^ ^]^ The potential drop at an electrolyte/channel interface is partitioned across two in series capacitors, associated to the areal channel interfacial capacitance *C*_*DL*,*ch*_ and to the areal chemical capacitance $C_{q,ch}\;\left(\Delta n\right)=\frac{\Delta n\cdot e^{2}}{\Delta \phi_{ch}\left(\Delta n\right)}\;$(termed quantum capacitance in layered 2D materials) that embodies the change Δϕ_*ch*_(Δ*n*) of the electrochemical potential of the organic semiconductor. This is expressed through the equation:

(4) $$\psi_{b}\left(V_{GS}\right)-\;V_{T}=\frac{\Delta \phi_{ch}\left(\Delta n\right)}{e}+\frac{\Delta n\cdot e}{C_{DL,ch\;}}$$

Plugging Equation (3) into Equation (4) yields:

(5) $$V_{GS}-\;V_{T}=\Delta n\cdot e\;\left[\frac{\Delta \phi_{ch}\left(\Delta n\right)}{\Delta n\cdot e^{2}}+\frac{1}{C_{DL\;}}\right]=\frac{\Delta n\cdot e}{C_{eff}\left(\Delta n\right)}$$

We recognize the areal interfacial capacitance $C_{DL\;}={\left[\frac{1}{C_{DL,ch\;}}+\frac{A_{Ch}}{A_{G}}\frac{1}{C_{G}}\right]}^{-1}$, the areal channel capacitance $C_{ch\;}\;\left(\Delta n\right)={\left[\frac{\Delta \phi_{ch}\left(\Delta n\right)}{\Delta n\cdot e^{2}}+\frac{1}{C_{DL,ch\;}}\right]}^{-1}$, and the areal effective capacitance of the whole EGOT $C_{eff}\;\left(\Delta n\right)=\frac{1}{A_{Ch}}\;\frac{A_{Ch}C_{ch\;}\left(\Delta n\right)A_{G}C_{G}}{A_{G}C_{G}+A_{Ch}C_{ch\;}\left(\Delta n\right)}$. Equation (5) expresses how the gate potential is partitioned across the EGOT device. To solve it for the areal charge carrier density Δ*n*, we need to express Δϕ_*ch*_(Δ*n*), whose functional form stems from the electronic structure of the organic semiconductor. The areal density of charge carriers Δ*n* will change according to the available electronic states in the semiconductor expressed as:

(6) $$\begin{array}{ccc} \Delta n & = & 2d\left[\int_{-\varepsilon }^{\varepsilon }dE\cdot f\left(E,\mu_{0}+\Delta \phi_{ch}\right)\mathrm{DOS}\left(E,\;\mu_{0}+\Delta \phi_{ch}\right)\right.\\ & & -\;\left.\int_{-\varepsilon }^{\varepsilon }dE\cdot f\left(E,\mu_{0}\right)\mathrm{DOS}\left(E,\;\mu_{0}\right)\right]\approx 2d\left[\int_{\mu_{0}}^{\mu_{0}+\Delta \phi_{ch}}dE\cdot \mathrm{DOS}\left(E\right)\right] \end{array}$$

The integration limit has been modified to finite ɛ because the DOS from Equation (1) is defined only in the bandgap. Here *f*(*E*, μ) is the Fermi population at energy E when the electrochemical potential is μ; the factor 2 accounts for spin multiplicity; *d* is the thickness of the semiconductor thin film. We apply Sommerfeld's lemma to get the equality on the right‐hand side, with the constraint that the integration interval [μ_0_; μ_0_ + Δϕ_*ch*_] ∈ [− ɛ ɛ].

In the following, it is convenient to renormalize the equations by means of the fractional variation of charge carriers $x=\frac{\Delta n}{2n_{max}\cdot d}\;,$ then rescaling by the energy disorder parameter σ the effective gate voltage $\nu =\frac{e(V_{GS}-V_{T})}{\sigma }=\frac{\mu_{0}}{\sigma }$, the half‐gap $\nu_{gap}=\frac{\varepsilon }{\sigma }=\frac{\Delta E_{gap}}{2\sigma }\;,$ and the electrochemical potential shift $\Delta \varphi =\frac{\Delta \phi_{ch}}{\sigma }\;,$ and introducing the ratio of areal charge densities $\alpha =(\frac{2e^{2}n_{max}d}{C_{DL}\sigma })$. Then, plugging Equations (1) and (2) into Equation (6) yields:

(7) $$x=\frac{2}{exp\left(\nu_{gap}\right)-exp\left(-\nu_{gap}\right)}\;\;\int_{\nu }^{\nu +\Delta \varphi }dx\cdot cosh\left(x\right)=\frac{sinh\left(\nu +\Delta \varphi \right)-sinh\left(\nu \right)}{sinh\left(\nu_{gap}\right)}$$

and the relation between Δφ and *x* reads:

(8) $$\;\left|\Delta \varphi \right|=sinh^{-1}\;\left\{x\;sinh\left(\nu_{gap}\right)+sinh\left(\left|\nu \right|\right)\right\}-\left|\nu \right|$$

**Figure 2:**
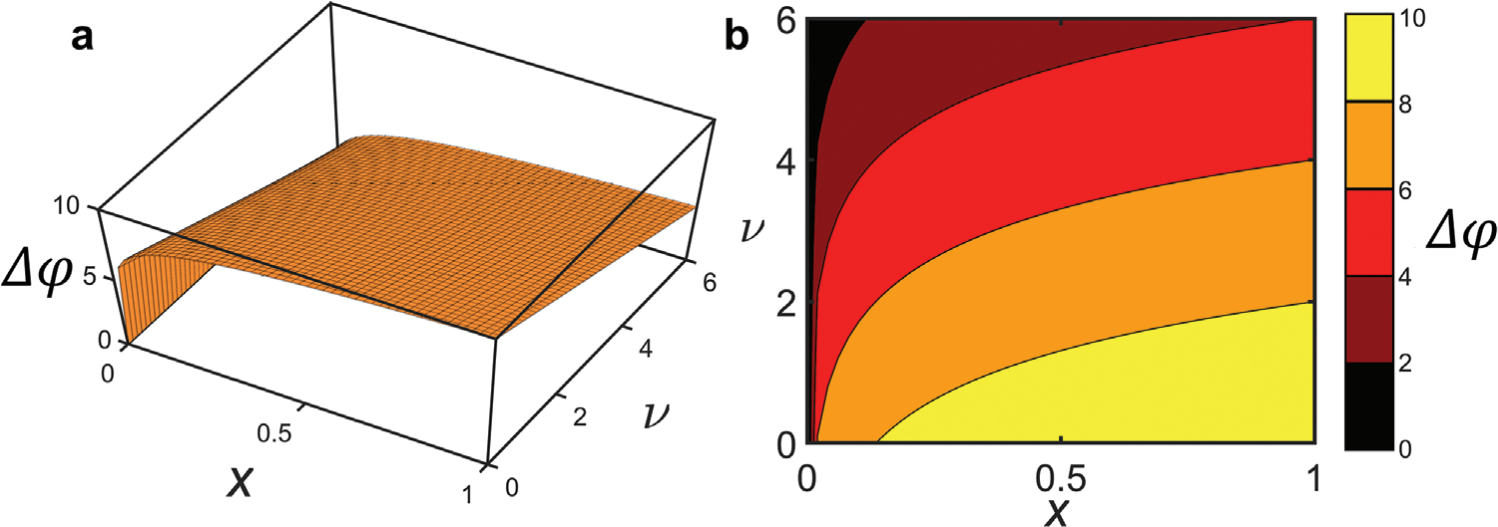
a) Plot of Δφ versus x and ν from Equation (8) for ν_gap_ = 10. b) contour plot of (a). The range of ν ∈ [0; ν_max_] is taken for n‐type semiconductor, for the p‐type will be symmetrical with inverted sign for Δφ.

Here *sinh^−1^* is the *inverse sinh* (or *arcsinh*) function. The inverse symmetry Δφ (− ν) = − Δφ(ν) allows us to focus only on either the first or third quadrant of (*x*, ν) relevant to an *n*‐type or *p*‐type semiconductor, respectively. We choose the first for **Figure** **2**. The shift of electrochemical potential Δφ is mostly sensitive to *x* when ν → 0 while when approaching the edges ν → ±ν_*gap*_, the shift Δφ weakly depends on *x*.

Plugging Equation (8) into Equation (5) yields the equation for the fractional charge carrier density *x*:

(9) $$\nu =\alpha x\left[\frac{sinh^{-1}\left\{x\;sinh\left(\nu_{gap}\right)+sinh\left(\nu \right)\right\}-\nu }{ax}+1\right]$$

**Figure 3:**
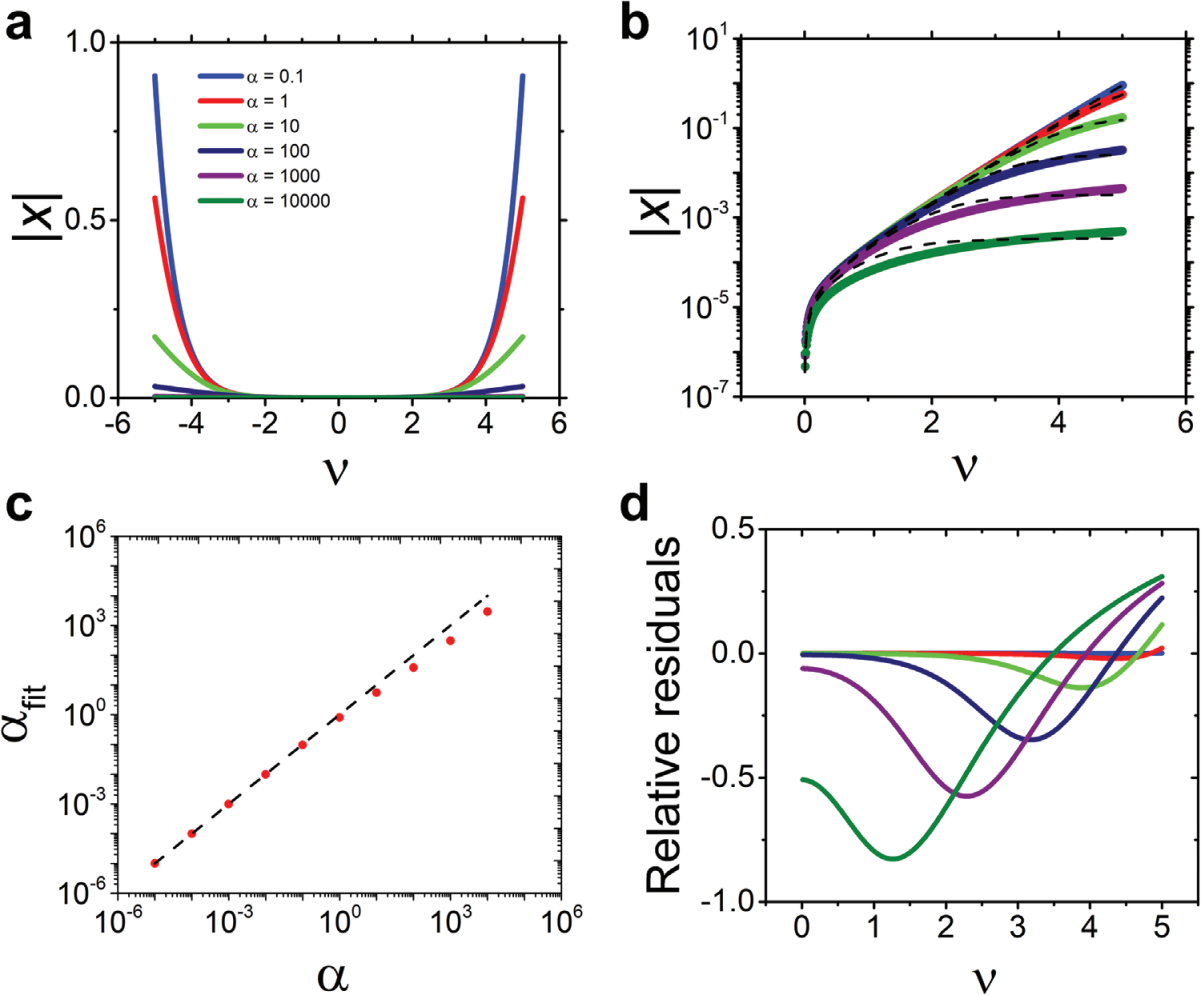
a) Plot of the numerical solution to Equation (10) |x| versus υ for different values of α and ν_gap_ = 10. b) same plot in log‐lin scale and comparison of the numerical solution to the best fit with Equation (11) for α from 0.1 to 10 000 (dashed lines). c) correlation plot between the values α_fit_ (red markers) versus α. The diagonal dashed line is correlation unity. The error bars of α_fit_ are equal or smaller than the marker's size. d) plot of relative residuals versus υ for different values of α.

The effective capacitance $\frac{C_{eff\;}}{C_{DL\;}}=\frac{\alpha x}{\nu }$ follows from Equation (9) as $\frac{C_{eff\;}}{C_{DL\;}}=\frac{\alpha x}{sinh^{-1}\left\{x\;sinh\left(\nu_{gap}\right)+sinh\left(\nu \right)\right\}-\nu +\alpha x}$. It depends on both the *C*_*DL*_ and the chemical capacitance $\frac{C_{q,ch}}{C_{DL\;}}=\frac{\alpha x}{\nu -\alpha x}=\frac{\alpha x}{sinh^{-1}\left\{x\;sinh\left(\nu_{gap}\right)+sinh\left(\nu \right)\right\}-\nu }\;$. The contribution of the latter to the effective capacitance is modulated by the charge carrier density *x* and ultimately by ν. Equation (9) is then recast as:

(10) $$x=\frac{sinh\left(2\nu -\alpha x\right)-sinh\left(\nu \right)}{sinh\left(\nu_{gap}\right)}$$

to express the nonlinear dependence of xvsυ and α. Equation (10) requires a numerical solution which is discussed in **Figure** **3**, then we analyze a few relevant approximations. Figure 3a shows the cross sections of the absolute value |*x*| at constant α values, evidencing the symmetry of the solution for holes and electrons and the characteristic “rectifier” shape of the curves. At the center of the ν range the fraction of carriers seems almost constant, the span of this range increasing at larger α values. This is also evident in the log‐lin plot in Figure 3b that highlights the power law dependence on ν and the smooth (logarithmic) dependence on the parameter α.

The dashed lines in Figure 3b are the result of an approximation that we infer as follows. We exploit the relations between the hyperbolic functions and their addition and multiple angle formulas^[^ ^27^ ^]^ to yield *x* *sinh* (ν_*gap*_) = *sinh*(2ν)[*cosh*(α*x*) − *sinh*(α*x*)] − *sinh*(α*x*)[*cosh*(ν) − *sinh*(ν)]^2^ − *sinh*(ν), then make the approximation for α|*x*| ≪ 1, viz. *cosh*(α*x*) ≈ 1 and *sinh*(α*x*) ≈ α*x*, to yield *x* *sinh*(ν_*gap*_) ≈ 2*sinh*(ν)*cosh*(ν)[1 − α*x*] − α*x* [*cosh*(ν) − *sinh*(ν)]^2^ − *sinh*(ν). It strictly holds at low carrier density at any α value, or for small α values. As we show later, most of the experimental data fulfil the latter condition, so the approximation holds in most of the real cases. We apply the duplication formulas for hyperbolic functions to obtain the approximate expression for *x* versus ν:

(11) $$x\;\approx \frac{sinh\left(2\nu \right)-sinh\left(\nu \right)}{sinh\left(\nu_{gap}\right)+\alpha \;cosh\left(2\nu \right)}$$

We assess the accuracy of Equation (11) by fitting the numerical solutions in Figure 3a for different values of α = 0.01 *to* 10^4^. The results are shown in Figure 3b as dashed lines, where the value of ν_*gap*_ is held fixed at the value 10, based on values of bandgaps 2 eV and energy disorder of 100 meV in organic semiconductors.^[^ ^28^, ^29^, ^30^ ^]^ The best fit curves (dashed lines) appear accurate across the whole set of exact data solutions (continuous lines), albeit there are deviations for the curves obtained at higher α values. Figure 3b also reveals that the *x* *vs* ν curves are weakly modulated by α for a large range of ν up to 3, when α < 100. This is the limit for breakdown of the initial hypothesis α|*x*| ≪ 1. In Figure 3c we compare the best fit value of α_*fit*_ from Equation (11) with the real value α, evidencing slight deviations at large α values. This is confirmed by the plot of the residuals normalized to the real value in Figure 3d, which exhibit the largest relative difference at low and high voltages on the order of ≈±0.5, else in absolute value of 0.01, again for large α values. The residuals are negligible when α values are small, as it is the case for a vast majority of application scenarios, thus the accuracy of Equation (11) is high throughout the ν range. Overall, Equation (11) accurately represents the charge carrier density across a large range of α < ν_*gap*_ and ν. This result hints also to another observation: the charge carrier density will correspond to the horizontal cross sections at constant α as in Figure 3a,b only when both disorder and interfacial capacitances stay constant. In real devices, for instance operated as affinity sensors, the interfacial capacitance often changes with the binding of biomolecules at the interface. Energy disorder may also change with the penetration of ions in the organic semiconductor channel. Thus, the parameter α may be sensitive to ambient conditions leading to *x* *vs* ν curves that interpolate across the curves shown in Figure 3a,b.

Another interesting approximation is for small values of |ν| ≪ 1, which allows us to expand Equation (8) as 2D Taylor series:

(12) $$\Delta \varphi \approx x\;sinh\left(\nu_{gap}\right)\left\{1-2\nu \;x\;sinh\left(\nu_{gap}\right)-x^{2}sinh^{2}\left(\nu_{gap}\right)+2\nu^{2}\text{…}\right\}$$

Equation (12) describes the relationship near the center of the HOMO‐LUMO gap in a regime of low doping. The leading term is *x* *sinh*(ν_*gap*_) which once plugged into Equation (5) yields $\frac{C_{eff\;}}{C_{DL\;}}=\frac{\alpha }{\alpha +sinh(\nu_{gap})}$. The effective capacitance, albeit gate‐voltage independent, contains a non‐negligible contribution from the chemical capacitance $\frac{C_{q,ch\;}}{C_{DL\;}}=\frac{\alpha }{sinh(\nu_{gap})}$ that depends on the bandgap of the organic semiconductor and will make the effective capacitance lower than the interfacial capacitance. For a large bandgap, *C*_*eff*_ ≈ *C*_*q*,*ch*_.

Another approximation is derived for |ν| ≫ 1, viz. the electrochemical potential is deep within the tail and the EGOT operates in a regime of high doping. Then Equation (7) becomes *x* ≈ − *exp*(− ν_*gap*_ − ν)[*exp*(− Δφ) − 1] ≤ 0 for holes, with ν≤0 and Δφ ≤ 0_;_ and *x* ≈ *exp*(− ν_*gap*_ + ν)[*exp*(Δφ) − 1] ≥ 0 for electrons with ν≥0 and Δφ ≥ 0. These equations are encompassed by the following expression:

(13) $$x\approx sgn\left(\nu \right)exp\left(-\nu_{gap}+\left|\nu \right|\right)\left[exp\left(\left|\Delta \varphi \right|\right)-1\right]$$

which establishes the expression for Δφ:

(14) $$\Delta \varphi \approx sgn\left(\nu \right)ln\left\{sgn\left(\nu \right)\;x\;exp\left(\nu_{gap}-\left|\nu \right|\right)+1\right\}$$

Equation (14) introduces a further boundary condition: the maximum electron areal density increase that may be induced in the tails of the organic semiconductor material is |*x*_max_| = 1 for |ν| = ν_*gap*_. Therefore, the maximum variation of the electrochemical potential is |Δφ_*max*_| = *ln*2 ≈ 1.4. This condition represents a strongly doped organic semiconductor, or a conductor, which is relevant to OECTs. In this case, we insert Equation (14) into Equation (5) that becomes ν = *sgn* (ν) *ln* [1 + *x* *sgn* (ν) *exp* (ν_*gap*_ − |ν|)] + α*x*. The charge carrier density is:

(15) $$\;\left|x\right|=\frac{W_{n}\left(\alpha \;exp\left[\alpha \;exp\left(\left|\nu \right|-\nu_{gap}\right)-\nu_{gap}+2\left|\nu \right|\right]\right)}{\alpha }\;-exp\left(\left|\nu \right|-\nu_{gap}\right)$$

Here *W*_n_ is Lambert function,^[^ ^31^, ^32^ ^]^ thus the effective capacitance reads:

(16) $$\frac{C_{eff\;}}{C_{DL\;}}\approx \frac{1}{\left|\nu \right|}\left\{W_{n}\left(\alpha \;exp\left[\alpha \;exp\left(\left|\nu \right|-\nu_{gap}\right)-\nu_{gap}+2\left|\nu \right|\right]\right)-\alpha \;exp\left(\left|\nu \right|-\nu_{gap}\right)\right\}$$

## 4 Effective Capacitance of the Device

**Figure 4:**
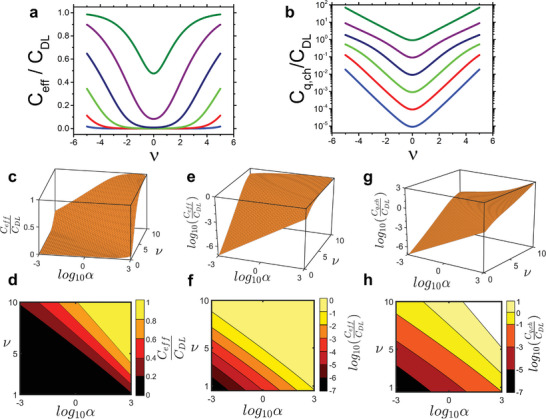
a) plot of the rescaled effective capacitance and b) chemical capacitance as a function of ν at several value of α. The charge density and the colors of the lines are the same as Figure 3a. c) rescaled effective capacitance and d) its contour plot versus scaled gate voltage ν and ratio of areal densities α from approximation Equation (17) high doping approximation; e,f) representation of panel (c) and panel (d) in Log scale; (g) and (g) rescaled chemical capacitance as from the approximation Equation (17) in Log scale.

We discuss now the behavior of the effective capacitance. In **Figure** **4**a we plot the rescaled $\frac{C_{eff\;}}{C_{DL\;}}$ using the numerical solution for *x* from Equation (10). The effective capacitance increases versus ν and tends asymptotically to the interfacial capacitance at large ν values. The effective capacitance changes from a minimum value at gate voltage near *V*_T_, where the chemical capacitance is sizable, to a maximum asymptotic value corresponding to the interfacial capacitance at large gate voltages. Both the minimum value and the slope around the minimum gets larger at increasing α. Thus, the modulation of the effective capacitance by the gate voltage increases for increasing α then slows down at very large α values as the effective capacitance becomes nearly equal to the interfacial capacitance. These large α values, however, are never observed in our experiments. For small α values, the apparent flatness of the effective capacitance hides the actual exponential increase of the dominant chemical capacitance around the minimum. This is shown with the trend of the rescaled chemical capacitance in Figure 4b. It is important to notice that in the low doping regime around the minimum, the chemical capacitance always contributes significantly to the effective capacitance across many orders of magnitude of the α values.

In Figure 4c,f we overlay the effective capacitance approximated with Equation (16) confirming its sensitivity to the gate voltage for high α values, for instance in presence of an ordered semiconductor films. Thus, our prediction is that for the same material a much greater gate voltage modulation will be observed in crystalline channels with respect to amorphous channels. The effective capacitance tends to the interfacial capacitance for large ν values approaching the band edge, hence in a regime of high doping. This also confirms that the chemical capacitance (Figure 4g,h) at high doping is the largest of the in‐series capacitors, so becomes negligible. At low voltage/low doping, on the other hand, the effective capacitance is few orders of magnitude smaller than the interfacial capacitance, and the chemical capacitance is dominant. According to the approximation made, both the effective and the chemical capacitances scale linearly with the logarithm of α at low ν values. In general, at low voltages ν and for α <10 (disordered semiconductor films) the effective capacitance is significantly contributed by the chemical capacitance. The low doping approximation $\frac{C_{q,ch\;}}{C_{DL\;}}=\frac{\alpha }{sinh(\nu_{gap})}\;$ is apparent from the linear profile in Figure 4g. The crossover from values $\frac{C_{q,ch\;}}{C_{DL\;}}$ < 1 to $\frac{C_{q,ch\;}}{C_{DL\;}}$ > 1 occurs, as expected, for increasing α and/or ν.

## 5 Analysis of the EGOT Transfer Characteristics

Once we have the effective capacitance, the EGOT current readily follows:

(17) $$I_{DS}\;\left(V_{GS};\;V_{DS}\right)=I_{DS,off}\;\left(\;V_{DS}\right)+\frac{W}{L}\mu_{h\left(e\right)}\;C_{eff\;}\;\left(V_{GS}-V_{T}\right)V_{DS}$$

where μ_*h*(*e*)_ is the hole(electron) charge carrier mobility, and the effective capacitance depends on *C*_*eff*_(*V*_GS_; *V*_T_; α; σ; ν_*gap*_). Due to the excellent agreement shown in Figure 3b between the numerical solution Equation (10) and the approximate analytical solution Equation (11), we substitute the latter in Equation (17) to yield:

(18) $$\begin{array}{c} I_{DS}\left(V_{GS};\;V_{DS}\right)\approx I_{DS,off}\left(V_{DS}\right)+\\ \left[\frac{W}{L}\mu_{h\left(e\right)}C_{DL}\;V_{DS}\right]\left\{\frac{\alpha \sigma }{e}\frac{sinh\left(2e\frac{V_{GS}-V_{T}}{\sigma }\right)-sinh\left(e\frac{V_{GS}-V_{T}}{\sigma }\right)}{sinh\left(\frac{\varepsilon }{\sigma }\right)+\alpha \;cosh\left(2e\frac{V_{GS}-V_{T}}{\sigma }\right)}\right\} \end{array}$$

that will be used to fit the experimental data. Equation (18) can be applied either to *p*‐type or *n*‐type devices, and also to ambipolar devices, upon replacing μ_*h*(*e*)_ with $\frac{1}{2}\left[\mu_{h}\left(1-sgn(V_{GS}-V_{T})\right)+\mu_{e}\left(1+sgn(V_{GS}-V_{T})\right)\right]$. It is apparent from Figure 3b that the voltage dependent Equation (18) will interpolate with continuity across the whole gate voltage range, thus providing a unified non‐discretionary tool for the analysis of the transfer curves from EGOTs without the need to assume either the type of device (EGOFET or OECT), or the segmentation into different response regimes. Thus, our model yields the accurate value of the switch‐on voltage V_T_. Interestingly, in Equation (18) the factor in square brackets is the linear transconductance $g_{m,l}=\frac{W}{L}\;\mu C_{DL}\;V_{DS}$, while the term in curly brackets is the effective voltage gating the organic semiconductor.

**Figure 5:**
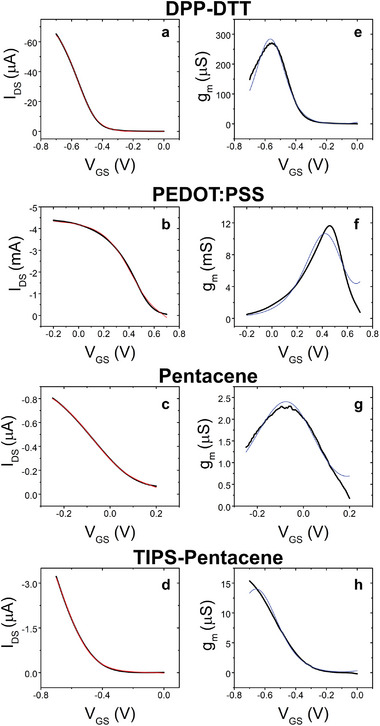
Experimental transfer curves (black lines) of EGOT based on a) DPP‐DTT, b) PEDOT:PSS, c) Pentacene, and d) TIPS Pentacene and the fitting curves from Equation (18) (red lines). The corresponding transconductance plots from the numerical derivative of the transfer curves (in black) and the fitting curves (in blue) for e) DPP‐DTT, f) PEDOT:PSS, g) Pentacene, and h) TIPS Pentacene. The electronic structure parameters and the best fit values are in Table 1.

**Figure 6:**
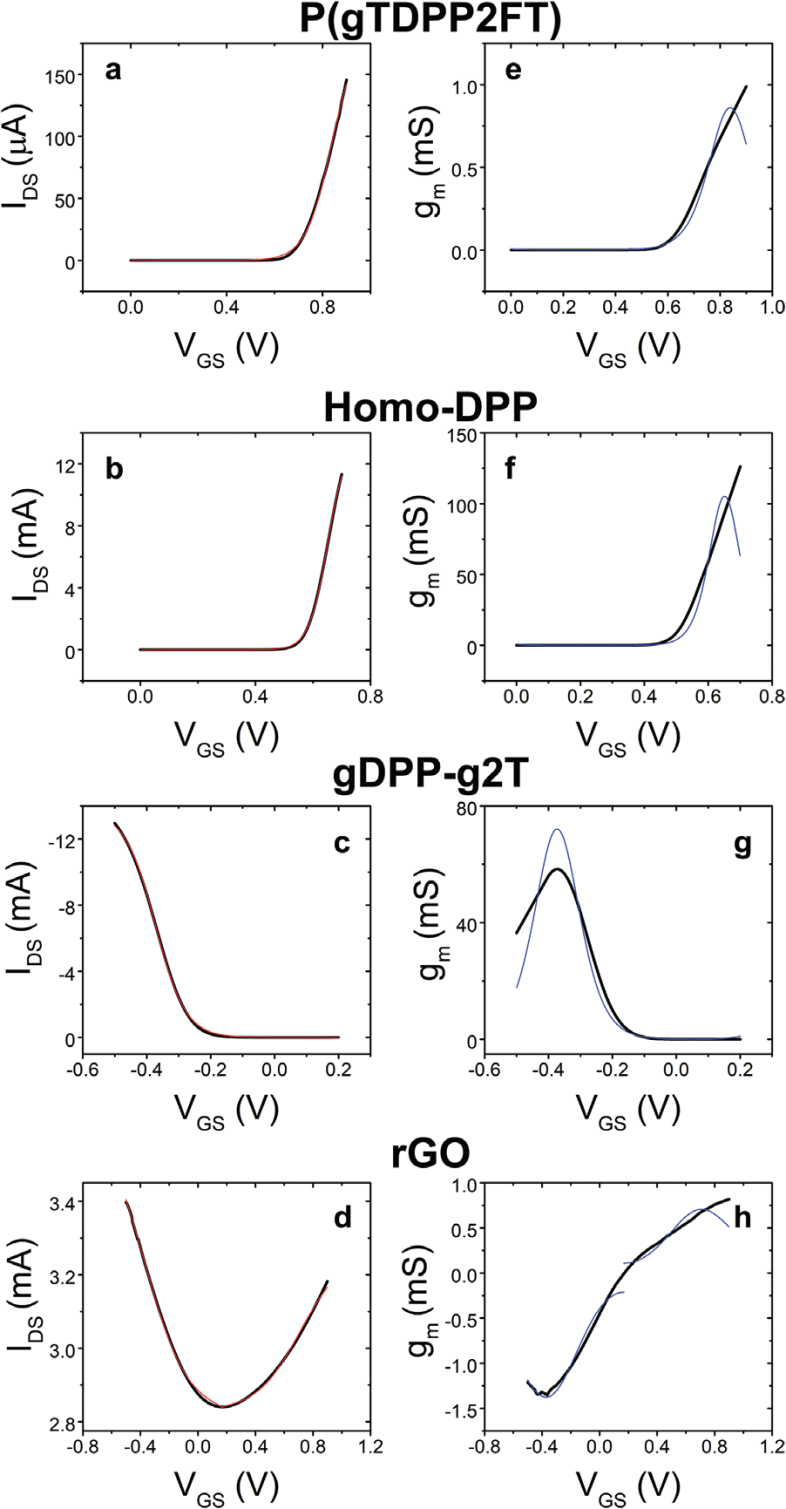
Experimental transfer curves (black lines) of EGOT based on a) P(gTDPP2FT), b) Homo‐DPP, c) gDPP‐g2T, and d) rGO and the fitting curves from Equation (18) (red lines). The corresponding transconductance plots from the numerical derivative of the transfer curves (in black) and the fitting curves (in blue) for e) P(gTDPP2FT), f) Homo‐DPP, g) gDPP‐g2T, and h) rGO. The electronic structure parameters and the best fit values are in Table 1. Data reported in panel (a,e) are from ref [41] data reported in panel (b,c,f,g) are from ref. [42].

To validate the model, we now challenge the current *I*_DS_ versus *V*_GS_ trend from Equation (18) fitting real experimental data. In **Figures** **5** and **6** we fit experimental transfer curves from EGOTs based on p‐type, n‐type and ambipolar semiconductors, from two different device architectures, viz. the classical top‐gate architecture and the recently developed vertical transistor architecture.^[^ ^33^, ^34^, ^35^, ^36^ ^]^ In **Figure** **5**a–d we report the experimental transfer curve and the fitting curve using Equation (18) with four variational parameters [*V*_T_; *g*_m,l_; *α*; *σ*] for p‐type (semi)conductive channels, namely Poly[2,5‐(2‐octyldodecyl)−3,6‐diketopyrrolopyrrole‐alt‐5,5‐(2,5‐di(thien‐2‐yl)thieno[3,2‐b]thiophene)] (DPP‐DTT) (5a), PEDOT:PSS (5b), Pentacene (5c), and 6,13‐Bis(tri‐isopropylsilylethynyl)pentacene (TIPS pentacene) (5d).

To perform the analysis, we fix the gap energy of each organic semiconductor according to their literature values, which are reported in **Table** **1**,^[^ ^37^, ^38^, ^39^, ^40^ ^]^ and the baseline off current *I*_*DS*,*off*_(*V*_DS_) with its mean value. In the case of PEDOT:PSS the off current in Equation (18) is substituted with the minimum of the current. Figure 5a–d shows the excellent agreement of the best fit curves to the experimental transfer curves. In Figure 5e–h we show the plots of the transconductance obtained by numerical differentiation of the experimental data, and the curves obtained by differentiation of the best fit curves from Equation (18). The latter test assesses the robustness of our fit also to the most stringent numerical differentiation. Interestingly, our fitted curve with TIPS Pentacene suggests that a maximum transconductance lies under the envelope of the numerically derived transconductance.

**Table 1:** Best fit parameters for the fit of current I_DS_ versus V_GS_ with Equation (18).

	DPP‐DTT	PEDOT:PSS	Pentacene	TIPS pentacene	P(gTDPP2FT)	Homo‐gDPP	gDPP‐g2T	rGO
ΔE_gap_ [eV]	1.7	1.7	2.2	1.82	1.36	1.08	1.08	0.5
σ [eV]	0.1271 ± 0.0003	0.204 ± 0.002	0.1895 ± 0.0006	0.189 ± 0.001	0.110 ± 0.002	0.0652 ± 0.0006	0.0951 ± 0.0009	0.319 ± 0.004
ν_gap_	6.69 ± 0.02	4.17 ± 0.04	5.80 ± 0.02	4.83 ± 0.03	6.2 ± 0.1	8.28 ± 0.07	5.68 ± 0.05	0.79 ± 0.01
α	0.72 ± 0.06	8.3 ± 0.4	27.6 ± 0.4	0.36 ± 0.03	0.011 ± 0.010	0.008 ± 0.004	0.05 ± 0.02	0.072 ± 0.002
V_T_ [V]	−0.118 ± 0.005	0.668 ± 0.002	0.1882 ± 0.0007	−0.100 ± 0.006	0.25 ± 0.05	0.23 ± 0.02	0.04 ± 0.02	0.168 ± 0.001
−*e*V_T_/σ	1.20 ± 0.04	−4.18 ± 0.06	−0.993 ± 0.007	0.67 ± 0.03	−2.3 ± 0.6	−3.5 ± 0.4	−0.5 ± 0.3	−0.67 ± 0.01
g_m,I_ (holes)	5.680·10^−4^ ± 7.0 ·10^−7^	2.13 10^−2^ ± 1.0 ·10^−4^	4.807·10^−6^ ± 6.0·10^−9^	2.80 10^−5^ ± 1.0 ·10^−7^	N/A	N/A	0.1441 ± 8.0 ·10^−4^	2.75 10^−3^ ± 2.0 ·10^−5^
g_m,I_ (electrons)	N/A	N/A	N/A	N/A	1.72 ·10^−3^ ± 2.0 ·10^−5^	0.210 ± 0.001	N/A	1.41 10^−3^ ± 1.0 ·10^−5^
Reduced Χ^2^	9.42 ·10^−14^	2.69·10^−9^	9.67 ·10^−18^	2.54 ·10^−16^	1.34 ·10^−12^	1.55 ·10^−9^	4.77 ·10^−9^	2.89 ·10^−11^
R^2^	0.99977	0.99885	0.99983	0.99968	0.99892	0.99979	0.99972	0.99869

In **Figure** **6**a–d we report the experimental transfer curve and the fitting curve for a polymeric n‐type (semi)conductive channels, namely P(gTDPP2FT) (6a),^[^ ^41^ ^]^ a vertical n‐type OECT fabricated with Homo‐gDPP (6b),^[^ ^42^ ^]^ a vertical p‐type OECT fabricated with gDPP‐g2T (6c),^[^ ^42^ ^]^ and reduced graphene oxide (rGO) (6d). While rGO transfer curve was recorded by us, the others come from Figure 3b of ref ^[^ ^41^ ^]^ (P(gTDPP2FT)) and Figure 3a (gDPP‐g2T) and 3c (Homo‐DPP) of ref ^[^ ^42^ ^]^. To perform the analysis, we fix the gap energy of each organic semiconductor according to their literature values, which are reported in Table 1,^[^ ^41^, ^43^, ^44^ ^]^ and the baseline off current *I*_*DS*,*off*_(*V*_DS_) with its mean value. In the case of rGO the off current in Equation (18) is substituted with the current at the charge neutrality point, viz. the absolute minimum of the transfer curve. Similarly to Figure 5 we also reported the derivative of the transfer and the fitting curves in Figure 6e–h.

It is remarkable how the model fits perfectly not only *p*‐type EGOFETs (DPP‐DTT, TIPS‐Pentacene), but also vertical and planar OECT (Homo‐gDPP, gDPP‐g2T and PEDOT:PSS), ambipolar rGO electrolyte gated transistors (EGT), and n‐type P(gTDPP2FT) device.

About the best fit values in Table 1, we notice that both the conditions of ν_*gap*_ ≫ 1, and small range of the voltage sweep are obeyed for all organic (semi)conductors; for rGO, ν_*gap*_ < 1, being the one with the smallest gap. The energy disorder parameter σ ranges from 0.1 to 0.4 eV for the materials chosen, the most disordered (semi)conductor being PEDOT:PSS. Again, rGO seems different with a larger disorder parameter. The parameter α ranges from 0.01 to 30 for the (semi)conductors, with the highest values exhibit by PEDOT:PSS, consistently with its large effective capacitance (as from Equation (21)) and transconductance. Interestingly, the same behavior can be observed in the case of pentacene devices since it is deposited through a highly controlled technique (vacuum deposition) that leads to a large number of available states for the charge carriers per unit of volume, similar to a crystalline structure. Conversely, the lowest limit is represented by the n‐type semiconductor, probably due to the lower number of available states for n‐type carriers in the condition where the OECTs work. Similarly, rGO exhibits a value α < 0.1. Vertical OECTs exhibit the highest transconductance, according to their geometrical features. While, talking about planar device, the transconductance is largest for PEDOT:PSS as the result of the large α. About the larger value of DPP‐DTT versus TIPS Pentacene, this may be correlated to the higher order (larger α and smaller σ). In the case of rGO, the transconductance for holes is about twice that for electrons, consistently with earlier findings.^[^ ^25^, ^26^, ^41^ ^]^ A final consideration concerns the fact that the DOS from Equation (1) is not customary for 2D materials as the energy dependence of the quantum capacitance is different.^[^ ^26^, ^45^, ^46^ ^]^ The good agreement of the fitted curve throughout the range may hint to a less‐than‐ideal 2D material with a behavior approaching that of a 3D disordered semiconductor. In brief, our model is sensitive and accurate, as it performs the analysis of the transfer curves throughout the whole gate voltage range, providing reasonable values of physical properties of the organic semiconductor material in the device. We highlight that this analysis does not require any ad hoc choice of data or parameters from the operator side.

We point out that our model does not rely on the usual threshold voltage parameter *V_th_* to describe the dependence of the output current *I*_DS_ to the applied potential *V*_GS_. The use of *V_th_* has been borrowed from conventional metal oxide‐semiconductor field‐effect transistors (MOSFETs) theory, but it poorly describes the physics of organic transistors, thus its definition is approximated as the onset voltage for the accumulation of charge carriers in the channel.^[^ ^47^, ^48^, ^49^ ^]^ Accordingly, *V_th_* is usually extracted with linear extrapolation methods.^[^ ^50^, ^51^ ^]^ Here, we treat *V_T_* as a fitting parameter that represents the gate voltage at which the (semi)conductor is in the flatband or charge neutrality condition. To demonstrate how *V_T_* differs from *V_th_*, as they describe different physical features, we quote the *V_th_* values extracted for the DPP‐DTT and TIPS Pentacene EGOTs reported in Figure 5: they are a few hundred mV more negative than the corresponding *V_T_* values reported in Table 1: indeed, *V_th_* values obtained from linear fit are −430 ± 1 mV for DPP‐DTT and −470 ± 1 mV for TIPS pentacene.

It is now clear that *V_T_*, which is sensitive to the functionalization of the gate electrode and/or the binding of molecules to the gate electrode or the channel, may also change $\frac{C_{eff}}{C_{DL}}$ by offsetting the rescaled gate voltage ν. However, unless the gate capacitance (which contributes in series to the interfacial capacitance *C_DL_* and hence to α) changes substantially, the smooth dependence on ν may not be straightforward to resolve experimentally. On the other hand, if both *V_T_* and interfacial capacitances change in sensing operations, our model predicts a crossover from small values of the rescaled effective capacitance at small α values, to large values at large α values. This scenario might be likely encountered, for instance, in organic transistor immunosensors with functionalized gates. Then, a stronger dependence of the effective capacitance on the gate voltage should be expected, which manifests into the shrinking of the linear region of the transistor response, as reported in previous works.^[^ ^3^ ^]^

Finally, we examine two limit cases of Equation (18). The first is for small values |ν| ≪ 1 for low electrostatic doping:

(19) $$I_{DS}\left(V_{GS};\;V_{DS}\right)\approx I_{DS,off}\left(V_{DS}\right)+\left[\frac{\alpha }{sinh\left(\frac{\varepsilon }{\sigma }\right)+\alpha \;}\right]g_{m,l}\left(V_{GS}-V_{T}\right)$$

In Equation (19) we truncate the series expansion to the linear term, neglecting higher odd powers of *V_GS_* − *V_T_*. For a large gap organic semiconductor ɛ ≫ σ, Equation (19) becomes:

(20) $$I_{DS}\approx I_{DS,off}+\frac{W}{L}\mu_{h\left(e\right)}V_{DS}\left\{d\left[\frac{4e^{2}\;n_{max}\;}{\sigma }exp\left(-\frac{\varepsilon }{\sigma }\right)\right]\right\}\left(V_{GS}-V_{T}\right)$$

The noticeable result is that the effective capacitance *C*_*eff*_ in curly brackets exhibits the linear scaling on thickness *d* as experimentally observed in OECTs.^[^ ^15^, ^16^ ^]^ Note that the interfacial capacitance *C*_*DL*_is now completely absent. This allows us to identify the term in square brackets in Equation (20), which exhibits the units of a capacitance per unit volume, as the volumetric capacitance C* of the organic semiconductor:

(21) $$\;C^{*}=\frac{4e^{2}\;n_{max}}{\sigma }\;exp\left(-\nu_{gap}\right)$$

Equation (21) could be rearranged as $C^{*}=2\alpha \frac{C_{DL}}{d}\;exp\;\left(-\nu_{gap}\right)$ to estimate the volumetric capacitance C* of our PEDOT:PSS device from the best fit values reported in I. The thickness of the PEDOT:PSS film is estimated by Atomic Force Microscopy  to be 19 ± 7 nm, while the values for C_DL_ are adopted from literature (1–10 µF cm^−2^). The calculated volumetric capacitance results between 0.09 and 2.3 F cm^−3^, thus lower than values reported in literature (6–60 F cm^−3^). The underestimation of the C* probably comes from the values of C_DL_ recorded in EGOT that do not consider the contribution of in series chemical capacitance. Another independent evaluation could be performed from the literature values of charge carriers mobility for PEDOT:PSS (i.e., between 0.1 and 1 cm^2^ V^−1^ s^−1^).^[^ ^52^, ^53^ ^]^ From the best fit values of the linear transconductance of our device we can extrapolate the real C_DL_ equal to 47 µF cm^−2^. These values lead to a calculated C* between 0.4 and 11 F cm^−3^ in a good agreement with reported values of C* in literature.^[^ ^15^ ^]^

We infer that the materials most suited for volumetric capacitance should exhibit a large density of states *n_max_* in the gap. The role of disorder is less intuitive, as on the one hand a large value of σ seems to decrease C*, on the other makes the parameter ν smaller as required in the approximation. Thus, we expect that both OECT and EGOFET in the voltage region around *V_T_* behave according to Equation (20) in the small voltage limit, with their effective capacitance scaling linearly with the film thickness.

The other limit is for 1<<|ν| ≈ ν_*gap*_ for high electrostatic doping. Then, the Taylor expansion of Equation (15) yields a voltage‐independent leading term $\frac{1}{2}<{\left(\frac{C_{eff\;}}{C_{DL\;}}\right)}_{\nu_{gap}}\approx \frac{1}{\nu_{gap}}\left\{W_{n}\left(\alpha \;exp[\alpha +\nu_{gap}]\right)-\alpha \right\}\leq 1$ whose value approaches unity as ν_*gap*_ is large. It turns out that the predicted current will be interfacial, albeit attenuated by the value of ${\left(\frac{C_{eff\;}}{C_{DL\;}}\right)}_{\nu_{gap}}$:

(22) $$I_{DS}\left(V_{GS};\;V_{DS}\right)\approx I_{DS,off}\left(V_{DS}\right)+g_{m,l}\;{\left(\frac{C_{eff\;}}{C_{DL\;}}\right)}_{\nu_{gap}}\left(V_{GS}-V_{T}\right)$$

In the “pure interfacial” limit ${\left(\frac{C_{eff\;}}{C_{DL\;}}\right)}_{\nu_{gap}}\to 1$ and the dependence on α and hence on the thickness *d* will disappear. Thus, this high doping limit yields the ideal EGOFET in the linear regime.

## 6 Conclusion

In conclusion, we proposed that in the electrolyte gated organic transistors the charge carrier density results from the in‐series combination of electrochemical and electrostatic capacitive coupling. The chemical capacitance, associated to ion flow in the organic semiconductor channel, significantly contributes to the effective capacitance, its weight being modulated by the gate voltage. The crossover from interfacial capacitance to chemical capacitance in the same device may occur, in principle, during the gate sweep. To assess it, we work out the solution to a model of an organic semiconductor with exponential energy disorder in the HOMO‐LUMO gap. The chemical capacitance is dominant at small gate voltages when the organic semiconductor is disordered. On the other hand, the effective capacitance approaches the interfacial capacitance for high gate voltages and ordered organic semiconductors (including for instance single molecular crystals), or for disordered materials when the number density of states in the gap is large. This view overcomes the limitations of the traditional models used either for EGOFET or OECT and unifies EGOFETs and OECTs into the same framework, the only difference being the weight of the chemical capacitance with respect to the interfacial capacitance. EGOFET and OECT are derived as limit cases of the gate voltage‐dependent effective (areal) capacitance. We show that the so‐called volumetric capacitance relates to the bandgap, max density of states and energy disorder of an organic semiconductor (**Table** **2** summarizes the adopted terminology and symbols).

**Table 2:** Symbols list.

Symbol	Definition
*σ*	Exponential decay rate of the DOS tail
$\alpha =(\frac{2e^{2}n_{max}d}{C_{DL}\sigma })$	Ratio between the maximum of charge carrier density in the DOS tail and the product of interfacial capacitance by the disorder factor
$\nu =\frac{e(V_{GS}-V_{T})}{\sigma }$	Rescaled gate bias relative to V_T_
$\;\nu_{gap}=\frac{\varepsilon }{\sigma }$	Rescaled bandgap
$x=\frac{\Delta n}{2n_{max}\cdot d}$	Rescaled charge carrier density
ɛ = Δ*E_gap_*/2	Half of bandgap
$\;c_{n}=\frac{1}{\left[1-exp(-\;\frac{2\varepsilon }{\sigma })\right]}\;\frac{n_{max}}{\sigma }$	Normalization constant
$\Delta \varphi =\frac{\Delta \phi_{ch}}{\sigma }$	Rescaled channel potential

An advantage of our model is that, even in the approximation Equation (18), enables the quantitative evaluation of the whole EGOT transfer curve, correctly reproducing the non‐linear voltage response. The validation against experimental data confirms the accurate prediction of the model on the physics of the device, showing the versatility of its use on unipolar and ambipolar devices either OECT or EGOFETs, and fostering its adoption for the quantitative analysis of experimental data in a very general framework deprived of arbitrary or phenomenological choices and interpretation on the data and the device type. We propose our current expression in Equation (18) as a useful tool to extract accurate and reliable values for the switch‐on voltage *V_T_*, the energy disorder and the bandgap of the organic semiconductor, as these important parameters are encompassed by our model.

## Conflict of Interest

The authors declare no conflict of interest.

## Data Availability

The data that support the findings of this study are available from the corresponding author upon reasonable request.
